# Verification of the detachment–transport coupling relationship of rill erosion using colluvium material in steep nonerodible slopes

**DOI:** 10.7717/peerj.14766

**Published:** 2023-01-24

**Authors:** Libo Chen, Pengyu Gao, Xiaolin Li, Qin Zhu, Zumei Wang, Fang Shuai, Yue Zhang, Jinshi Lin, Yanhe Huang, Fangshi Jiang

**Affiliations:** Jinshan Soil and Water Conservation Research Center, Fujian Agriculture and Forestry University, Fuzhou, China

**Keywords:** Colluvium deposit, Flume test, Rill flow, Steep slope, Water erosion

## Abstract

The detachment–transport coupling equation by Foster and Meyer is a classical equation that describes the relationship between detachment and transport. The equation quantifies the relationship between sediment loads and soil detachment rates, deepens the understanding of soil erosion and provides a reliable basis for the establishment of an erosion model. However, the applicability of this equation to slopes with gradients greater than 47% is limited. In this work, the detachment–transport coupling relationship is investigated using the colluvium material of Benggang. A nonerodible rill flume 4 m long and 0.12 m wide was adopted. The slope gradient ranged from 27% to 70%, the unit flow discharge ranged from 0.56 × 10^−3^ to 3.33 × 10^−3^ m^2^ s^−1^, and the sediment transport capacity (*T_c_*) was measured under each slope and discharge combination. The sediment was inputted into the flume according to the predetermined sediment addition rate (from 0% to 100% of *T_c_*), and the detachment rate (*D_r_*) under each combination of the slope and discharge was measured. *D_r_* linearly decreased with increasing sediment loads, which is consistent with the detachment–transport coupling equation by Foster and Meyer. The linear equations can predict the detachment capacity (*D_c_*) and *T_c_* well (Nash–Sutcliffe efficiency coefficient (NSE) = 0.98 for *D_c_*, and NSE = 0.99 for *T_c_*). The detachment–transport coupling equation can adequately predict the *D_r_* (NSE = 0.89). However, its applicability to slopes of <47% (NSE: 0.92–0.96) was greater than that to slopes of ≥47% (NSE: 0.81–0.89), and the predicted *D_r_* under *T_c_* levels of 20% and 40% were higher than the measured values, while the predicted value under a *T_c_* level of 80% was lower than the measured value. In summary, the detachment–transport coupling equation by Foster and Meyer can accurately reflect the negative feedback relationship between detachments and transports along steep-slope fixed beds and is suitable for colluvial deposit research. The results provide a basis for the construction of steep-slope colluvial deposit erosion models. In the future, the study of the hydrodynamic characteristics of sediment transport processes should be strengthened to clarify the detachment–transport effect of flows through hydrodynamics.

## Introduction

Soil erosion occurs when topsoil is displaced due to exogenous forces. At present, erosion has become a serious global environmental problem that not only causes water and soil loss but can also cause losses in soil nutrients, water pollution and the drying of lakes and ponds (Boardman, 2006; Zhao et al., 2013; Orimoogunje, 2014). Soil erosion processes caused by runoff or rainfall can be subdivided into three interrelated subprocesses: soil detachment, transport, and deposition. Detachment–transport coupling is the most widely used concept based on these three subprocesses (Wang, Flanagan & Engel, 2019). This concept was used in the Water Erosion Prediction Project (WEPP) model (Flanagan & Nearing, 1995) to quantify the effect of the sediment load on the soil detachment rate. The detachment–transport coupling concept can be intuitively explained by the first-order detachment–transport coupling equation of Foster & Meyer (1972), which models detachment as a function of the deficit sediment transport capacity (Knapen et al., 2007) as follows:

(1) $$\displaystyle{{{{D}_{r}}} \over {{{D}_{c}}}}+\displaystyle{{{{q}_{s}}} \over {{{T}_{c}}}} {\;= 1}$$

where *D*_*r*_ is the detachment rate of rill water flow, *D*_*c*_ is the detachment capacity of rill water flow, *q*_*s*_ is the sediment load of water flow, and *T*_*c*_ is the sediment transport capacity of rill water flow. After shifting, Eq (1) can be expressed as:

(2) $${Dr={ D_c(1-\frac{q_s}{T_c})}}$$

Eq. (2) can be further expressed as:

(3) $$D_{\rm r}=a-{\rm b}q_s$$

where a = *D*_*c*_, b = *D*_*c*_/*T*_*c*_, and a/b = *T*_*c*_. Nearing et al. (1990) described the detachment–transport coupling equation (Foster & Meyer, 1972) as a straight line connecting two endpoints, with one end representing the detachment capacity given a sediment content of zero and the other end representing the sediment transport capacity given a detachment rate of zero; notably, the soil detachment rate linearly decreases with an increasing sediment transport rate. The detachment–transport coupling equation (Foster & Meyer, 1972) is based on observation and mathematical hypothesis without experimental verification, and its accuracy under different experimental conditions must be verified.

Scholars have verified the rationality of the detachment–transport coupling concept through fixed-bed (nonerodible bed) or movable bed (erodible bed) experiments. In terms of movable bed experiments, Cochrane & Flanagan (1997) simulated rill runoff by using a flume with a constant slope and found that sediment addition caused soil detachment to decrease. Equation (3) can be applied under all test conditions. Merten, Nearing & Borges (2001) chose two sizes of a homogeneous glass material and injected this material from the upper part of a flume to simulate two modes of detachment rate reduction, *i.e*., turbulence reduction and trench bottom protection. The results revealed that the detachment rate decreased with increasing sediment loads, and the detachment rate followed a first-order function of the sediment load. Lei et al. (2002) studied the relationship between the detachment rate and sediment load by simulating different rill lengths and observed that the detachment rate linearly decreased with an increasing sediment load; moreover, the results confirmed the validity of the detachment–transport coupling equation. Zhang, Li & Ding (2005) used four types of rare earth elements (REEs) to obtain sediment loads following detachment at different rill locations. Based on along-rill erosion data, their work confirmed the WEPP detachment–transportation coupling concept and its rationality. According to the sediment load varying along eroding rills, Zhang et al. (2014) verified that the first-order coupling equation under high discharge on steep slopes can be used to express the relationship between the sediment load and detachment rate in loess rills. Govers, Giménez & Van Oost (2007) summarized studies related to the detachment–transport coupling concept and noted that the effect of the sediment load on the detachment process could not be independently studied, which made it difficult to interpret the experimental results. Therefore, scholars (Zhang et al., 2009; Shen, Wang & Wang, 2016; Shen et al., 2017) have reported that it is possible to add sediment at the top of a fixed bed and detach it in a specific region near the bottom to independently study the effect of the sediment load on detachment. Zhang et al. (2009) verified the negative feedback effect of the sediment load on the detachment rate in sediment-laden flow under various slopes and discharge combinations with riverbed sediment as a sediment source, and Shen, Wang & Wang (2016) obtained the same results using loose soil *via* a similar experimental method. These two studies also showed that the detachment capacity, sediment transport capacity and detachment rate could be accurately predicted through Eq. (3) under these experimental conditions.

Conversely, not all findings could support the detachment–transport coupling equation (Foster & Meyer, 1972). Meyer & Wischmeier (1969) proposed the concept whereby detachment and sediment transport are two independent processes, and sediment erosion is limited by the lesser of the two. The field experimental results of Huang, Laflen & Bradford (1996) supported this concept. Giménez & Govers (2002) noted that when detachment and sediment transport are controlled by different hydraulic parameters, detachment and sediment transport processes cannot be simply linked, as indicated in the detachment–transport coupling equation (Foster & Meyer, 1972). Lei et al. (2006) tracked the dynamic process of rill erosion using REE tracers. Their results showed that the effect of the sediment load on the detachment rate did not occur under all slope conditions. Under low slopes (5° and 10°), the sediment load linearly increased with the rill length, and the sediment load did not affect or slightly affected the detachment rate, indicating that the relationship between sediment transport and detachment may be affected by the slope. Sirjani & Mahmoodabadi (2014) claimed that discharge may also affect the relationship between the sediment load and detachment rate. Other studies have shown that the sediment load exerts no or little effect on the detachment rate (Govers, Giménez & Van Oost, 2007; Polyakov & Nearing, 2003). Additionally, the abovementioned research mainly considered slope gradients lower than 47% (25°). However, slopes with gradients above 47% are common on the colluvial deposits in Benggang erosion area, and the runoff hydrodynamics generated under these gradients differ from those on slopes with gradients lower than 47% (Jiang et al., 2018a; Zhan et al., 2020). Thus, under the condition of high gradients and steep slopes (>47%), the applicability of the detachment–transport coupling equation (Foster & Meyer, 1972) must be further verified.

**Figure 1:**
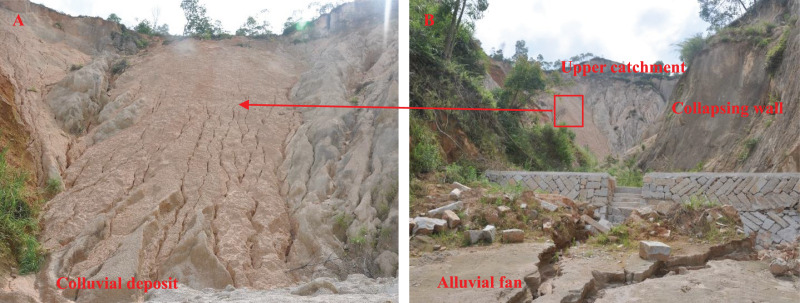
A typical Benggang in the study area. (A) Loose colluvial deposits with many rills. (B) Upper catchment, collapsing wall, colluvial deposit and alluvial fan.

Benggang refers to a kind of erosion landform established *via* sediment collapse driven by the combined actions of hydraulic power and gravity (Jiongxin, 1996; Jiang et al., 2018b), which is common in Southeast China (Liao et al., 2019; Xia et al., 2019). The erosion process causes damage to local farmland and considerable economic losses. According to research statistics, the soil erosion modulus in Benggang landforms is tens of times higher than that on gentle slopes (Jiongxin, 1996; Zhong et al., 2013). A typical Benggang erosion landform mainly comprises four parts, namely, an upper catchment, collapsing wall, colluvial deposit and alluvial fan (Fig. 1) (Jiongxin, 1996), among which the colluvial deposit is mainly established *via* the accumulation of collapsing wall collapse sediment at the foot of the slope. The colluvial deposit exhibits a loose structure and high slope gradients (generally between 36% and 84%), and there is a large amount of colluvium material on it. This material is very susceptible to rainfall- and runoff-induced erosion and is the main sediment source in Benggang erosion (Jiang et al., 2014). Many scholars have studied the runoff, sediment transport and hydrodynamic characteristics of colluvial deposits (Jiang et al., 2018a; Zhan et al., 2020; Jiang et al., 2018b; Lin et al., 2017). Lin et al. (2017) found that the average velocity and unit flow power could be used as suitable predictors of the sediment transport capacity in movable bed experiments. Through a simulated rainfall experiment, Jiang et al. (2018b) found that the unit flow power was the best hydraulic parameter that characterizes the rill erosion rate, and the fixed-bed flume experimental results showed that the modified velocity was the best predictor of the sediment transport capacity (Jiang et al., 2018a). Zhan et al. (2020) found significant correlations between the sediment transport capacity and the slope and between the discharge and gravel content.

The above studies show that the existence of a detachment-transport coupling mechanism in the process of soil erosion is still controversial. Additionally, few works have been reported regarding the detachment-transport concept on steep colluvial deposit slopes, which limits a greater understanding of the erosion processes of colluvial materials. Therefore, the purpose of this study is to verify the applicability of the detachment–transport coupling equation (Foster & Meyer, 1972) for steep-slope colluvium materials using a fixed-bed flume experiment to avoid the effect of deposition (Shen, Wang & Wang, 2016) while addressing three objectives: (1) quantifying the soil detachment rate in response to the sediment load, (2) to analysing the simulation accuracy of the sediment transport and detachment capacities, and (3) to analysing the simulation accuracy of the soil detachment rate.

## Materials and Methods

### Study area

Experimental soil was obtained from Yangkeng Village of Longmen Township, Anxi County (24°57′ N, 118°05′ E), which experiences a subtropical monsoon climate with an average annual precipitation of 1,800 mm and an average annual temperature of 19 °C. The soil in the study area is developed from acidic medium- and coarse-grained granite, and the rock minerals mainly include quartz and feldspar. Due to the combination of sufficient water and high temperatures, the rocks in the study area weathered to form a thick weathering crust with a thickness of tens of metres exhibiting a high content of coarse particles, a loose structure and weak soil erosion resistance. Under the influence of human activities, the main vegetation in the study area comprises *Pinus massoniana* and *Eucalyptus grandis×E. urophylla*.

The understorey vegetation largely includes *Dicranopteris dichotoma*, *Eriachne pallescens*, *Syzygium buxifolium*, and other species (Zhang et al., 2022; Zhou et al., 2022). Due to the low soil erosion resistance and human-made destruction of vegetation, Benggang landforms are easily generated in the study area under the combined action of hydraulic power and gravity. According to a field survey, there are 125,828 Benggang units in Anxi County, accounting for approximately 50% of the total number of Benggang units in Fujian Province. Among the various regions, the small Yangkeng Village (5.6 km^2^) contains a total of 226 Benggang units, with a density of 40 units per km^2^, which is 10 times that in Anxi County and 200 times that in Fujian Province. Therefore, the Benggang development area in Yangkeng Village is one of the representative Benggang areas in Anxi County and a typical area of granite Benggang development in southern China (Jiang et al., 2014; Zhang et al., 2022; Zhou et al., 2022).

### Experimental soil sample

Soil samples were collected from the typical Benggang colluvium materials of the colluvial deposit in Yangkeng Village of Longmen Town, Anxi County, and the samples were evenly mixed after air drying. After the colluvium material sediment samples were naturally dried, the physical and chemical properties of the sediment were analysed.

The pH value of the soil (<2 mm), which had a soil‒water ratio of 1 to 2.5, was measured through a pH metre (STARTER 2100, OHAUS Instruments Co., Ltd., Shanghai, China) to be 5.19. The organic matter content was determined with a wet oxidation method (Bernoux & Cerri, 2005) and was 1.68 g kg^−1^. The particle size was determined through a QICPIC particle instrument from New Patek Company in Germany. Grain size analysis revealed that the gravel (2–10 mm), sand (0.05–2 mm), silt (0.002–0.05 mm) and clay (<0.002 mm) contents were 30%, 45%, 23%, and 2%, respectively, and the median grain size diameter (*D*_*50*_) was 0.79 mm.

### Experimental equipment

**Figure 2:**
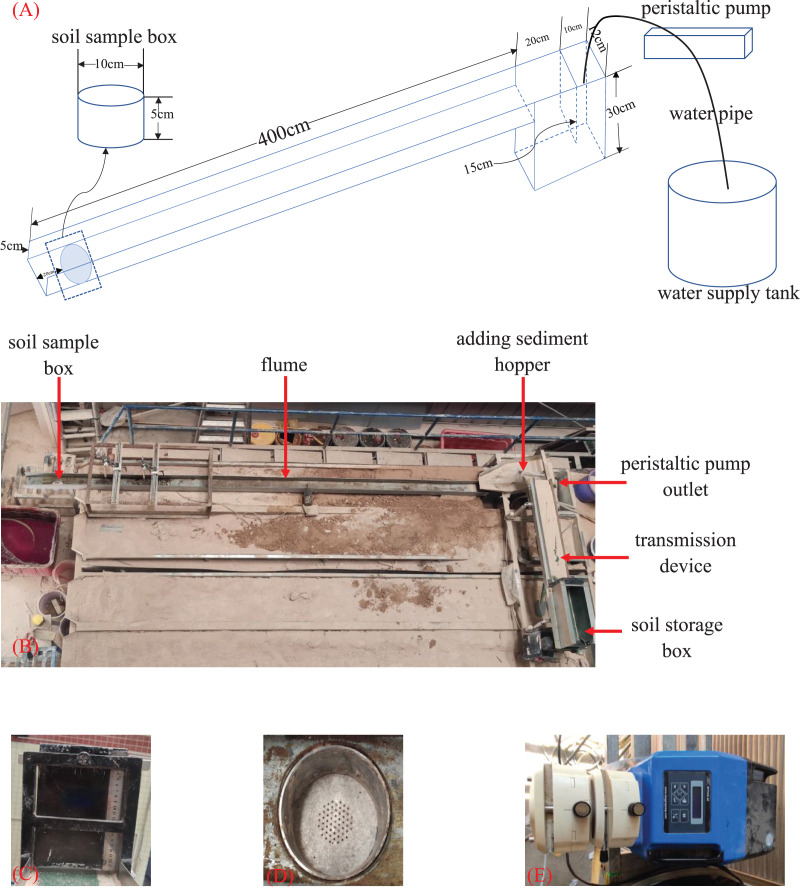
Experimental equipment. (A) A schematic diagram of the flume and water supply system. (B) The experimental device. (C) The baffle of a sediment storage box,that controls the sediment supply rate by lifting the height. (D) The sediment sample box, diameter 10 cm, deep 5 cm. (E) The peristaltic pump.

The experimental equipment comprises three components: a water supply system, rill flume and sediment supply system (Fig. 2). Before the experiment was performed, a layer of test sediment (particle size <5 mm) was stuck to the bottom and along both sides of the flume to ensure that the roughness of the flume bottom and sides was similar to that of the test surface and gully walls, respectively.

The water supply system included a water supply tank, peristaltic pump, overflow tank, and other components. The water supply tank had a capacity of 10 m^3^ to provide water during the test. The peristaltic pump (WT600-4F-C, Longer Precision Pump Co., Ltd., Hebei, China) provided a consistent water flow rate during the test and used controlled dual pump heads to maintain a pre-set discharge level. The size of the overflow tank was 0.3 m × 0.12 m × 0.3 m, and a 0.12 m × 0.15 m partition was embedded in the middle of the overflow tank. The top of the partition was flush with the top of the flume, and the bottom of the partition was suspended at a height of 0.15 m to ensure that water could freely circulate in the two separated flumes to achieve a steady flow effect.

The sediment supply system included a transmission device and sediment hopper. The transmission device comprised a transmission belt, the speed of which was controlled by a motor, and a baffle was installed on the side of the sediment storage box close to the hopper to control the sediment discharge height to achieve an accurate and constant sediment supply rate. The lower end of the sediment hopper was parallel to the flume so that the sediment sample could be uniformly added to the flume.

According to the field survey, the average rill width on the slope of the colluvium material is approximately 12 cm, so the length, width and height of the rill flume were set to 4, 0.12 and 0.1 m, respectively. The bed slope of the flume could be adjusted from 0 to 84%. A soil sample box was located 0.2 m from the bottom of the flume, which could be embedded with a circular ring knife with an inner diameter of 9.8 cm and a height of 5.0 cm. The bottom was evenly drilled to facilitate the discharge of water under gravity.

### Experimental design

According to the results of previous studies on the same subjects (Jiang et al., 2014; Lin et al., 2017), combined with the frequency of rainstorms in the study area and the runoff unit flow discharge generated on the colluvial deposit slope, a total of four discharge gradients were established. These four discharge gradients were equivalent to unit flow discharges of 0.56 × 10^−3^, 1.11 × 10^−3^, 2.22 × 10^−3^, and 3.33 × 10^−3^ m^2^ s^−1^. According to the field investigation results, the slope of colluvial deposits could reach as high as 40°. Meanwhile, five slope gradients were designed: 27%, 36%, 47%, 58% and 70%. The slope and discharge values were combined, and the sediment transport capacity (*T*_*c*_) was measured under each combination. Then, the detachment rates under sediment transport capacities of 0% *T*_*c*_, 20% *T*_*c*_, 40% *T*_*c*_, 60% *T*_*c*_, 80% *T*_*c*_ and 100% *T*_*c*_ were measured.

### Experimental procedures

The air-dried soil was divided into two parts: one part was used as the sediment supply source in the sediment transport test; the other part of the soil sample was sealed for 48 h under a controlled moisture content of approximately 15%. After the water content had stabilized, soil was added to the circular ring knife (with an inner diameter of 9.8 cm and a height of 5.0 cm). Before soil was added to the circular ring knife, a layer of gauze was placed flat across the bottom of the ring knife so that the test soil within the ring knife could evenly absorb water and become saturated. In the process of soil addition, the soil bulk density was controlled at approximately 1.40–1.45 g cm^3^ (Zhang et al., 2022), and the ring knife containing the soil sample was soaked in water for 24 h before use.

Before the test, the steel trough was adjusted to the pre-set slope, and a peristaltic pump was used to iteratively adjust the test discharge to the pre-set discharge level (with a discharge variability less than 20 mL min^−1^). The saturated soil sample was left standing for 1 h without absorbing water so that water could drain under gravity. Then, the soil sample was placed in the soil sample box at the bottom of the flume, and the surface of the soil sample was levelled to match the bottom of the flume and covered with a thin iron sheet to ensure that the surface was flush with the flume. Before sediment addition, the water flow was stabilized, after which sediment was added to the flume using the system described above. The sediment supply rate was controlled such that as the water flow transported the sediment, there remained a small amount of sediment deposited on the flume bed. In the sediment addition process, the position of the sediment supply port was adjusted to fully disperse the sediment so that the water flow transported the sediment in a saturated sediment load state. When water was initially discharged from the flume outlet into the sink, the thin iron sheet was removed, and five water–sediment mixture samples were collected at the outlet. Sediment load data under the different combinations of the given discharge and slope were obtained through drying and weighing (Jiang et al., 2018a; Zhan et al., 2020). Each group of experiments was repeated three times for a total of 60 repeated experiments. The sediment load obtained at this time was determined as the sediment transport capacity under the current combination of discharge and slope conditions, and a zero-detachment rate was assumed.

Sediment was inputted into the flume according to the predetermined sediment addition rate. When the soil detachment depth in the test ring knife reached approximately 2 cm (Zhang, 2002), water and soil addition was halted, and the flushing time was recorded. The remaining sediment in the test ring knife, after scouring, was dried and weighed, and the water flow detachment rate was calculated based on the weight of the test sediment sample (Zhang et al., 2009; Shen, Wang & Wang, 2016; Shen et al., 2017). The experiments were repeated three times considering each combination of the various slope and discharge conditions and sediment supply rates for a total of 300 repeated experiments. The soil detachment rate in clear water was considered the detachment capacity under this combination of slope and discharge conditions (Nearing et al., 1989).

### Experimental parameters and model accuracy index

The sediment transport capacity of rill flow can be obtained as the product of the maximum sediment load of rill flow and the discharge:

(4) $${T_c={{\rm q}c_{max}}}$$

where *T*_*c*_ is the sediment transport capacity of rill flow (kg m^−1^ s^−1^), q is the unit flow discharges (m^2^ s^−1^), and *c*_*max*_ is the maximum sediment load achieved by rill flow during the actual test (kg m^3^).

The rill flow detachment rate can be calculated as follows:

(5) $${Dr = }\displaystyle{{{\rm Ww - Wd}} \over {{\rm tA}}}$$

where *D*_*r*_ is the water flow detachment rate (kg m^−2^ s^−1^), Ww is the dry sediment weight before the test is performed (kg), Wd is the dry sediment weight after the test is performed (kg), t is the water flushing time (s), and A is the sediment box area (m^2^). Under clear water conditions, the soil detachment rate (*D*_*r*_) equals the soil detachment capacity (*D*_*c*_).

The relative error (RE), mean relative error (MRE), mean absolute relative error (MARE), coefficient of determination (R^2^), and Nash–Sutcliffe efficiency coefficient (NSE) were considered to analyse the model accuracy; these terms can be calculated as follows:

(6) $${\rm RE = }\displaystyle{{{{\rm P}_{\rm i}}{\rm - }{{\rm O}_{\rm i}}} \over {{{\rm O}_{\rm i}}}}{\rm \times 100\% }$$

(7) $${\rm MRE = }\displaystyle{{\rm 1} \over {\rm n}}\mathop \sum \nolimits_{{\rm i = 1}}^{\rm n} \displaystyle{{{{\rm P}_{\rm i}}{\rm - }{{\rm O}_{\rm i}}} \over {{{\rm O}_{\rm i}}}}{\rm \times 100\% }$$

(8) $${\rm MARE = }\displaystyle{{\rm 1} \over {\rm n}}\mathop \sum \nolimits_{{\rm i = 1}}^{\rm n} \left| {\displaystyle{{{{\rm P}_{\rm i}}{\rm - }{{\rm O}_{\rm i}}} \over {{{\rm O}_{\rm i}}}}} \right|{\rm \times 100\% }$$

(9) $${{\rm R}^{\rm 2}}{\rm = }\displaystyle{{{{\left[ {\mathop \sum \nolimits_{{\rm i = 1}}^{\rm n} \left( {{{\rm O}_{\rm i}}-{ \bar {\rm O}}} \right)\left( {{{\rm P}_{\rm i}}-{\bar {\rm P}}} \right)} \right]}^{\rm 2}}} \over {\mathop \sum \nolimits_{{\rm i = 1}}^{\rm n} {{\left( {{{\rm P}_{\rm i}}-{\bar {\rm P}}} \right)}^{\rm 2}}\mathop \sum \nolimits_{{\rm i = 1}}^{\rm n} {{\left( {{{\rm P}_{\rm i}}-{\bar {\rm P}}} \right)}^{\rm 2}}}}$$

(10) $${\rm NSE = 1 - }\displaystyle{{\sum {{\left( {{{\rm O}_{\rm i}}{\rm - }{{\rm P}_{\rm i}}} \right)}^{\rm 2}}} \over {\sum {{\left( {{{\rm O}_{\rm i}}-{\bar {\rm P}}} \right)}^{\rm 2}}}}$$

where O_i_ is the measured value of the sample i, P_i_ is the predicted value of the sample i,Ō is the average measured value,$\bar P$ is the average predicted value, and n is the sample number. For NSE ≥0.7, the model performs well; for NSE <0.7, the model performance is adequate; and for NSE <0.4, the model performance is poor.

### Statistical analysis

Excel 2016 was used for data statistics, and RE, MRE, MARE, R^2^, NSE were calculated according to their corresponding formulas. Origin 2018 was used to draw images. SPSS 26 was used to fit the equations.

## Results and discussion

### Variation in the soil detachment rate with the sediment load

**Figure 3:**
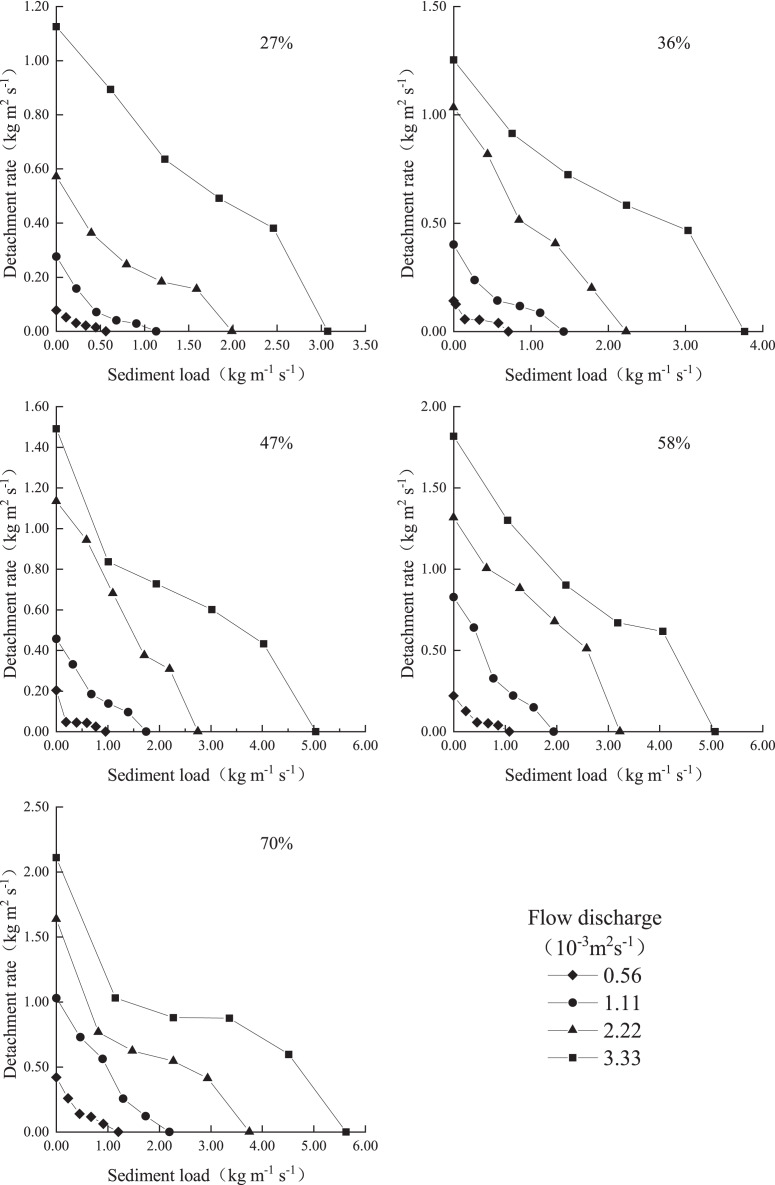
Variation in the detachment rate with the sediment transport rate under the different discharge and slope conditions.

The colluvium material detachment rate decreased with increasing sediment load under each condition (Fig. 3). Under a sediment load of zero, the detachment rate was the highest, *i.e*., the soil detachment capacity was attained. When the sediment load reached 100% (*i.e*., the maximum sediment load), the detachment rate was zero, *i.e*., the runoff sediment transport capacity was attained. The above suggests that soil detachment exerts a negative feedback effect on sediment transport; notably, the sediment in water flow inhibits the detachment effect of water flow on the sediment. These results are consistent with the results of previous studies (Zhang et al., 2009; Shen, Wang & Wang, 2016; Shen et al., 2017). Figure 3 further shows that under the same slope, the curves reflecting the detachment rate variation with the sediment load exhibited similar slopes overall, but uneven changes could be observed at different positions of each curve, which occurred because the hydraulic characteristics of the different sediment-laden flows varied. The soil detachment rate tended to decrease with increasing sediment load. There are three common viewpoints to explain these findings. First, under certain discharge and slope conditions, the flow energy remains constant, and there exists a trade-off relationship between the energy used for sediment transport and that used for detachment (Zhang et al., 2009; Moore & Burch, 1986). Second, turbulent flow causes soil detachment, and an increase in the sediment load inhibits water flow turbulence (Wang & Larsen, 1994; Nearing & Parker, 1994; Escauriaza & Sotiropoulos, 2011). Third, sediment fulfils a protective role that can reduce the contact area between the water flow and sediment, thereby decreasing detachment (Merten, Nearing & Borges, 2001; Polyakov & Nearing, 2003).

Table 1 provides the regression equation between the rill flow detachment rate and sediment load under each condition. The table indicates that the relationship between the rill flow detachment rate and sediment load satisfied Eq. (3), the R^2^ value for each regression analysis result ranged from 0.83 to 1.00, and the NSE value was greater than 0.83 (>0.70), indicating that the equation reached statistical significance and could sufficiently describe the relationship between the rill flow detachment rate and sediment load under the experimental conditions. The equation form is consistent with the first-order linear coupling relationship proposed for detachment–transport coupling (Foster & Meyer, 1972). The linear negative feedback effect of the sediment load on the soil detachment rate according to the first-order linear coupling relationship was validated by Zhang et al. (2009) using Haplesterv and riverbed sediment under slope gradients between 8.8% and 47% and by Shen, Wang & Wang (2016) involving loess under slope gradients between 10.5% and 38.4%. In our study, we considered slope gradients between 27% and 70%, which are much higher than the highest slope gradients in the above experiments (Zhang et al., 2009: 47%; Shen, Wang & Wang, 2016: 38.4%). It could be verified that the linear negative feedback effect of the sediment load on the soil detachment rate also occurred under slope gradients between 47% and 70%.

**Table 1:** Relationship between the detachment rate and sediment load of colluvium material slopes under the different combinations of slope and discharge conditions.

Slope gradient (%)	Discharge (m^2^ s^−1^)	Simulation equation *D*_r_ = a − b*q*_s_	Measured detachment capacity (kg m^−2^ s^−1^)	Predicted detachment capacity a (kg m^−2^ s^−1^)	Measured sediment transport capacity (kg m^−1^ s^−1^)	Predicted sediment transport capacity a/b (kg m^−1^ s^−1^)	R^2^	NSE
27	0.56 × 10^−3^	*D*_r_ = 0.069 − 0.129*q*_s_	0.078	0.069	0.558	0.535	0.94	0.94
27	1.11 × 10^−3^	*D*_r_ = 0.224 − 0.227*q*_s_	0.276	0.224	1.132	0.987	0.86	0.84
27	2.22 × 10^−3^	*D*_r_ = 0.507 − 0.255*q*_s_	0.572	0.507	1.989	1.988	0.93	0.93
27	3.33 × 10^−3^	*D*_r_ = 1.109 − 0.340*q*_s_	1.125	1.109	3.074	3.262	0.98	0.98
36	0.56 × 10^−3^	*D*_r_ = 0.120 − 0.167*q*_s_	0.142	0.120	0.708	0.719	0.83	0.92
36	1.11 × 10^−3^	*D*_r_ = 0.341 − 0.250*q*_s_	0.401	0.341	1.424	1.364	0.91	0.94
36	2.22 × 10^−3^	*D*_r_ = 0.996 − 0.453*q*_s_	1.034	0.996	2.236	2.199	0.98	0.99
36	3.33 × 10^−3^	*D*_r_ = 1.208 − 0.293*q*_s_	1.254	1.208	3.767	4.123	0.95	0.98
47	0.56 × 10^−3^	*D*_r_ = 0.172 − 0.202*q*_s_	0.203	0.172	0.957	0.851	0.85	0.84
47	1.11 × 10^−3^	*D*_r_ = 0.413 − 0.247*q*_s_	0.457	0.413	1.743	1.672	0.95	0.97
47	2.22 × 10^−3^	*D*_r_ = 1.145 − 0.411*q*_s_	1.134	1.145	2.750	2.786	0.99	0.99
47	3.33 × 10^−3^	*D*_r_ = 1.304 − 0.249*q*_s_	1.490	1.304	5.036	5.237	0.91	0.95
58	0.56 × 10^−3^	*D*_r_ = 0.184 − 0.184*q*_s_	0.220	0.184	1.083	1.000	0.88	0.91
58	1.11 × 10^−3^	*D*_r_ = 0.769 − 0.420*q*_s_	0.828	0.769	1.944	1.831	0.95	0.97
58	2.22 × 10^−3^	*D*_r_ = 1.322 − 0.365*q*_s_	1.317	1.322	3.229	3.622	0.95	0.98
58	3.33 × 10^−3^	*D*_r_ = 1.712 − 0.321*q*_s_	1.816	1.712	5.078	5.333	0.96	0.98
70	0.56 × 10^−3^	*D*_r_ = 0.358 − 0.339*q*_s_	0.419	0.358	1.141	1.056	0.91	0.94
70	1.11 × 10^−3^	*D*_r_ = 0.979 − 0.484*q*_s_	1.028	0.979	2.167	2.023	0.98	0.99
70	2.22 × 10^−3^	*D*_r_ = 1.445 − 0.395*q*_s_	1.636	1.445	3.658	3.666	0.93	0.91
70	3.33 × 10^−3^	*D*_r_ = 1.943 − 0.335*q*_s_	2.110	1.943	5.639	5.800	0.95	0.92

In erodible beds, certain studies (Cochrane & Flanagan, 1997; Merten, Nearing & Borges, 2001; Lei et al., 2002) have verified the existence of a detachment–transport coupling relationship by the variation in the detachment rate or sediment load along the flow path. It was found that with increasing slope length, the sediment load of water flow continued to increase, and the soil detachment rate gradually decreased. Moreover, when the sediment load reached saturation, the detachment rate reached zero. Huang, Laflen & Bradford (1996) and Lei et al. (2006) obtained a linear relationship between the sediment load and slope length, and the detachment–transport coupling relationship was not considered to exist. This may occur because the erodible bed soil erosion process is detachment-limited and cannot sufficiently supply sediment (Zhang, 2017), which results in a constant detachment rate, while the sediment load linearly increases with the flow path. Of course, this may also occur because the detachment–transport coupling relationship could not be determined due to the limited slope length (Lei et al., 2001) in the experiments of Huang, Laflen & Bradford (1996) and Lei et al. (2006). The above demonstrates that the detachment–transport coupling relationship for erodible beds is still disputed based on soil erodibility, hydrodynamic properties and other conditions (Zhang, 2017), and further research is needed.

### Simulation accuracy analysis of the sediment transport and detachment capacities

To further assess the coupling concept between the detachment rate and sediment load proposed by Foster & Meyer (1972), the detachment and sediment transport capacities were predicted *via* linear regression (Table 1) and compared to the measured data. According to the analysis of the various evaluation indices of the model error (Table 2), the RE between the predicted and measured values of the sediment transport capacity was −12.2%, the MRE was 1.0%, the MARE was 5.4%, the R^2^ value was 0.99, and the NSE value was 0.99. This indicates that the transport capacity could be accurately simulated with the detachment–transport coupling equations for rill flow on colluvial slopes under the different slopes and discharge combinations.

**Table 2:** Statistics of the measured and predicted data using the detachment–transport coupling equation.

Parameter	RE	MRE	MARE	R^2^	NSE	*n*
*T*_c_	−12.2% to 11.1%	1.0%	5.4%	0.99	0.99	20
*D*_c_	−1.0% to 16.4%	9.0%	9.8%	0.99	0.98	20
*D*_r_	−355.0% to 48.4%	−27.7%	38.0%	0.92	0.89	80

According to the analysis of the various evaluation indices of the model error (Table 2), the RE between the predicted and measured values of the soil detachment capacity was −1.0%, the MRE was 16.4%, the MRE was 9.0%, MARE was 9.8%, the R^2^ value was 0.99, and the NSE value was 0.98. These results show that the detachment capacity could also be accurately determined with the established detachment–transport coupling equations for rill flow on colluvial slopes under the different slopes and discharge combinations.

**Figure 4:**
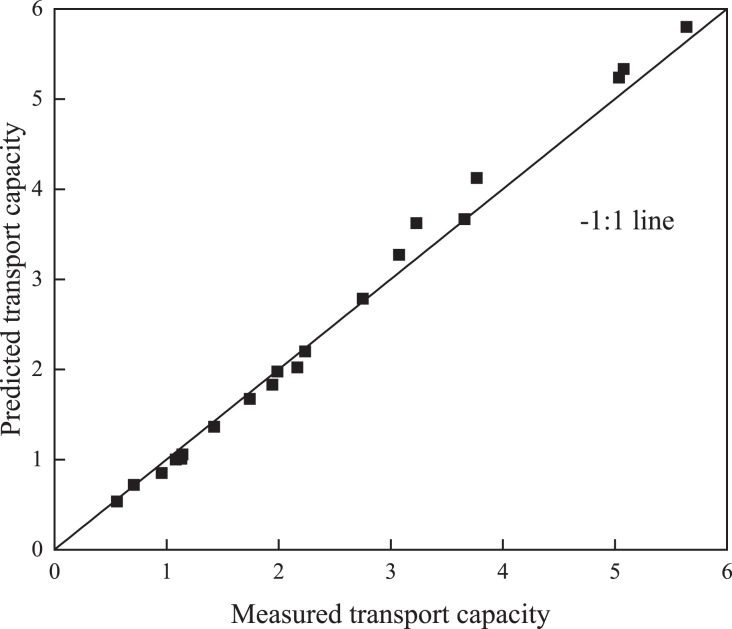
Comparison between the measured and predicted transport capacity values.

**Figure 5:**
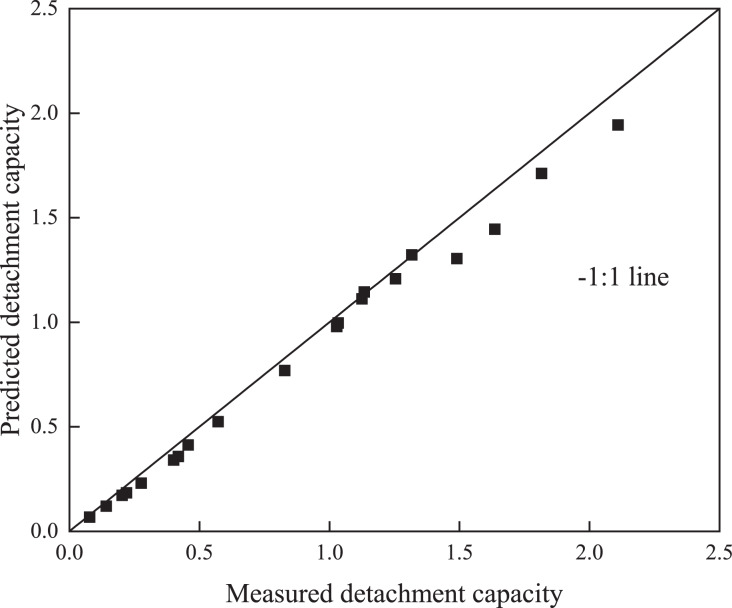
Comparison between the measured and predicted detachment capacity.

In the comparison between the measured and predicted values (Figs. 4 and 5), the points in both figures were closely spaced around the 1:1 line, which is consistent with the findings obtained in Table 2. The results obtained by both Zhang et al. (2009) and Shen, Wang & Wang (2016) also indicated that detachment and sediment transport capacities could be predicted with the detachment–transport coupling equation (Foster & Meyer, 1972).

It has also been found that sediment transport capacities and detachment capacities can be obtained from erodible bed tests (Lei et al., 2001; Chen et al., 2016; Lei et al., 2002). The sediment load and rill length models were constructed based on the concept of detachment-transport coupling, and the sediment transport capacity of the flow path was obtained by Lei et al. (2001) and Chen et al. (2016). However, Polyakov & Nearing (2003) predicted sediment transport capacities from both the erosional side (by continuous erosion along a rill) and the depositional side (by deposition of excess sediment) and found that the maximum sediment load obtained from the erosional side was only half of that obtained from the depositional side. It is considered that the hydrodynamic state of sediment-laden flow also needs to be considered for water flow sediment transport capacities. Soil detachment capacity refers to the soil detachment rate under clear water conditions, so the detachment rate at the entrance of the rill is used to represent the detachment capacity in the detachment rate and rill length model constructed by Lei et al. (2002). However, Giménez & Govers (2002) concluded that the role of hydrodynamic parameters needs to be considered when using this method to simulate the detachment capacity due to the variable hydrodynamic properties along the flow path. In summary, the prediction of sediment transport capacities and detachment capacities in erodible bed tests by the detachment-transport coupling equation needs to be studied in depth.

### Simulation accuracy analysis of the detachment rate

According to the measured detachment capacity, sediment transport capacity and sediment load, Eq. (3) was used to obtain the predicted values of the soil detachment rate under the different conditions for a comparison to the corresponding measured data. Table 2 demonstrates that the measured and predicted soil detachment rates suitably agreed (R^2^ = 0.92 and NSE = 0.89), the MRE value was −29.1%, and the MARE value was 39.3%. However, compared to the results provided in Table 2, the fitting accuracy of the sediment transport and detachment capacities was superior to that of the soil detachment rate. Zhang et al. (2009) also obtained the same conclusion. Equation (1) requires the same experimental materials for the determination of *D*_*c*_ and *T*_*c*_. Although the same material composition was maintained throughout this experiment, the structure differed. Scattered and loose colluvium was used in the sediment transport experiments, while colluvium with a certain bulk density (1.40–1.45 g cm^−3^) was used in the soil detachment experiments. These two experimental materials exhibited different structures, and thus, there were certain differences that could impact the detachment rate results, yielding a lower accuracy than that of the *D*_*c*_ and *T*_*c*_ results.

Statistical analysis was conducted on the measured and predicted soil detachment rate values under the different slope gradients (Table 3). Table 3 reveals that with an increasing slope gradient, the values of RE, MRE, MARE, R^2^, and NSE did not exhibit regular changes. The NSE value was the lowest under a slope gradient of 70%, but the value was still higher than 0.7, indicating that Eq. (1) remains applicable under a slope of 70%. Although there occurred no regular change between the NSE and the slope gradient, as listed in Table 3, the detachment rate prediction model accuracy coefficient (NSE) under the higher slope gradients (slopes between 58% and 80%) was lower than that under the lower slope gradients (slopes between 27% and 36%). This result indicates that the equation was less applicable for slopes above 25° because the resulting water flow was more unstable under the higher slope gradients (Zhang et al., 2010; Zhao et al., 2015), which resulted in an increased experimental uncertainty.

**Table 3:** Statistics of the soil detachment rate measurement and prediction data under the different slope conditions using the detachment–transport coupling equation.

Slope (%)	RE	MRE	MARE	R^2^	NSE	*n*
27	−354.9% to 45.7%	−37.1%	46.0%	0.961	0.957	16
36	−99.2% to 47.6%	−15.0%	27%	0.929	0.921	16
47	250.8% to 31.0%	−42.6%	51.1%	0.899	0.843	16
58	122.3% to 48.4%	−17.0%	32.4%	0.896	0.886	16
70	−84.2% to 29.2%	−26.7%	33.5%	0.930	0.814	16

Statistical analysis was also performed on the measured and predicted soil detachment rate values under the different unit flow discharge conditions (Table 4). Table 4 indicates that with an increasing unit flow discharge, the values of RE, R^2^, and NSE did not exhibit regular changes. However, with an increasing unit flow discharge, there was a downwards trend in the MRE and MARE. This occurred because the measured value was lower under a low unit flow discharge, the accuracy requirements were extremely high, and the measurement results at this time were prone to multiple errors affecting the model results (Shen et al., 2017), which also yielded an NSE value of only 0.07 when the unit flow discharge was 0.56 × 10^−3^ m^2^ s^−1^. When the unit flow discharge was increased from 1.11 × 10^−3^ m^2^ s^−1^ to 3.33 × 10^−3^ m^2^ s^−1^, the NSE value exhibited a downwards trend, which could also be attributed to the instability in sandy water flow under high discharge levels (Zhang et al., 2010; Zhao et al., 2015). Additionally, the NSE values were generally lower under the unit flow discharge conditions provided in Table 4 than those listed in Table 3. This may occur because in sediment-laden flow, the dominant factor of the change in the soil detachment rate is the unit flow discharge (Shen et al., 2019), which could lead to the low accuracy of statistical analysis based on this factor as the classification standard.

**Table 4:** Statistics of the soil detachment rate measurement and prediction data under the different discharge conditions using the detachment–transport coupling equation.

Discharge (m^2^/s)	RE	MRE	MARE	R^2^	NSE	*n*
0.56 × 10^−3^	−250.8% to 33.6%	−61.3%	64.7%	0.83	0.07	20
1.11 × 10^−3^	−354.9% to 5.2%	−48.8%	49.4%	0.94	0.82	20
2.22 × 10^−3^	−48.6% to 48.4%	−2.94%	18.9%	0.83	0.75	20
3.33 × 10^−3^	−42.5% to 47.6%	−2.20%	18.8%	0.91	0.62	20

**Figure 6:**
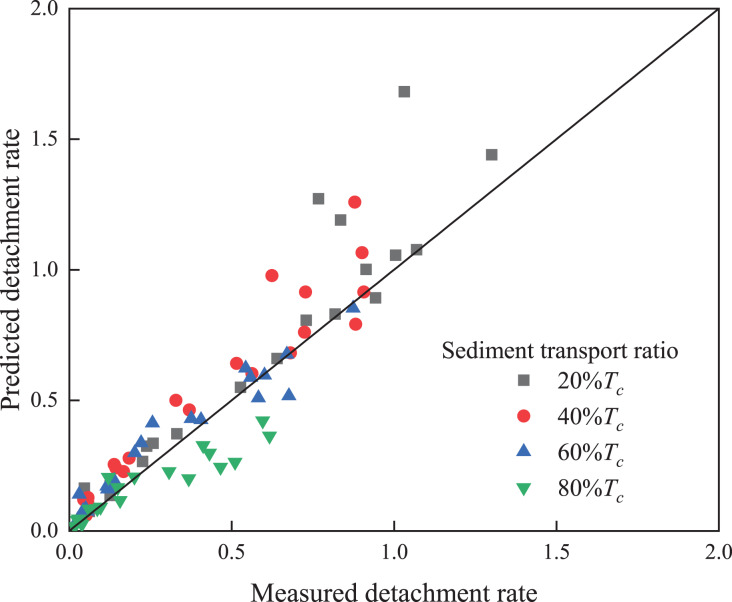
Comparison of the measured and predicted values of the soil detachment rate under the different sediment transport ratios.

Comparative analysis of the measured and predicted values of the soil detachment rate under the different sediment load conditions (Fig. 6) also revealed that under *T*_*c*_ values of 20% and 40%, the predicted detachment rate was higher than the measured value. As shown in Fig. 3, under a low sediment load, the trend line exhibited a concave shape. The detachment–transport coupling equation (Foster & Meyer, 1972) was established based on the interaction between water and sediment. The ratio between the sediment load and sediment transport capacity defines the relative transport energy term, and the ratio between the detachment rate and detachment capacity defines the relative detachment energy term. The sum of these two ratios is one, *i.e*., the total available energy. According to the theory presented in detachment–transport coupling by Foster & Meyer (1972), the water flow energy is only used for evacuation and detachment, and the sediment load thus increases; then, the detachment amount decreases according to the corresponding input proportion. However, in actual research, it was found that after the addition of sediment to clear water under certain slope and discharge conditions, the viscosity coefficient of water flow increased, the drag coefficient increased, and the Froude and Reynolds numbers accordingly decreased (Zhang et al., 2010). In addition to transporting sediment, sediment-laden flow must overcome the interaction among sediment particles, which may cause energy dissipation (Zhang et al., 2010). Moreover, the sediment in sediment-laden water flow reduces the contact area between the water flow and sediment and inhibits detachment. Therefore, when the sediment load increases according to a certain proportion, the energy used by the water flow for soil detachment is not proportionally reduced but is less than this proportion, resulting in an actual detachment amount that is smaller than the predicted detachment amount (Merten, Nearing & Borges, 2001; Polyakov & Nearing, 2003).

As shown in Fig. 6, most of the 80% *T*_*c*_ points occurred below the 1:1 line, with some occurring very far from the 1:1 line. This phenomenon is also observed in Fig. 3. When the sediment load approached the sediment transport capacity, the trend line exhibited a convex shape, which may be mainly related to the characteristics of a high sediment load in the water flow. The uneven distribution of sediment and local turbulence in water under high sediment loads causes the sediment-laden flow to detach more sediment (Shen, Wang & Wang, 2016; Liu, Zhang & Han, 2008). Liu, Zhang & Han (2008) and Shen, Wang & Wang (2016) also reported that a small fraction of the sediment was still detached under saturated sandy water flow conditions.

## Conclusions

In this experiment, Benggang colluvium material obtained in southern China was employed as the study object to assess the detachment–transport coupling equation in describing the detachment–transport coupling concept under steep-slope conditions (slope gradients higher than 47%). The research results revealed that the detachment rate achieved a negative feedback relationship with the sediment load, and the relationship between these two quantities could be represented as a linear function, which is consistent with the detachment–transport coupling equation. This equation could sufficiently simulate the detachment and sediment transport capacities under the different experimental conditions. The detachment–transport coupling equation was more suitable for slopes with gradients lower than 47% than for slopes with gradients higher than or equal to 47%. In addition, flow and sediment load could affect the predictive capability of the detachment–transport coupling equation in regard to the soil detachment rate. In conclusion, the detachment–transport coupling equation can be suitably adopted for colluvial deposits with steep slopes, and the obtained results can deepen the understanding of the mechanism of the colluvial deposit erosion process. Furthermore, by analysing the response of the detachment rate to the sediment load under different slope and discharge combinations, this study found that they roughly accord with the first-order coupling equation, rather than a completely linear relationship. Thus, it could be seen that the detachment rate was not only affected by the sediment load. In this experiment, the sediment load of the flow was high, and the sediment particles would inevitably affect the hydrodynamic characteristics of the flow. Therefore, the study of the hydrodynamic characteristics of the sediment transport process should be strengthened to clarify the detachment–transport effect of flow through hydrodynamics to provide a basis for perfecting the erosion theory of soil‒rock mixed media.

## Supplemental Information

## Additional Information and Declarations

### Data Availability

The following information was supplied regarding data availability: The raw measurements are available as a Supplemental File.
